# Management of severe tibial bony defects with double metal blocks in knee arthroplasty—a technical note involving 9 cases

**DOI:** 10.3109/17453674.2010.548031

**Published:** 2011-02-10

**Authors:** Seung-Wook Baek, Choong H Choi

**Affiliations:** Department of Orthopaedic Surgery, Hanyang University College of Medicine, South Korea

Severe bony defects during either primary or revision knee arthroplasty are common. If the defect is > 10 mm in its greatest depth, metal augmentation or a bone graft should be considered. We report the operative technique of metal augmentation using double metal blocks for severe uncontained proximal tibial defects.

## Surgical technique and postoperative protocol

**Figure 1.**
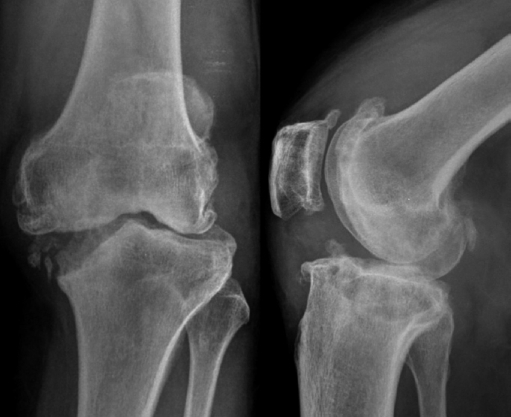
Preoperative radiographs with marked medial tibial bone loss.

**Figure 2.**
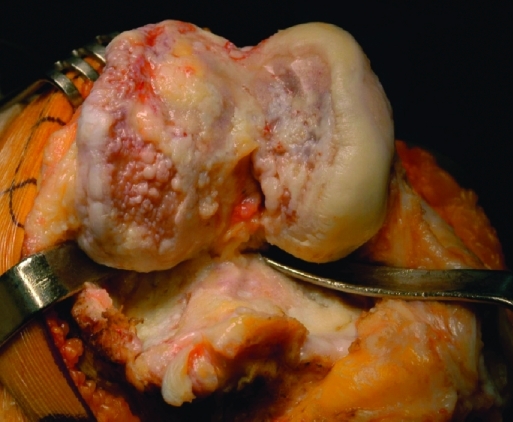
Intraoperative photograph after arthrotomy.

Through an anterior midline skin incision and medial parapatellar approach, the knee joint is exposed (Figures 1 and 2). Soft tissue is released to obtain balancing of varus or valgus deformities. The intramedullary alignment instrumentation is used to prepare the femoral side.

The extramedullary tibial alignment guides are applied to the tibia. Once proper axial alignment is verified, the proximal end of the tibia can be cut at usually 10 mm depth from the unaffected tibial condyle. The tibial surface is then prepared to accept the tibial base tray. The sclerotic base of the defect is cut to expose a flat, cancellous bony surface and the concave, irregular defect is converted to a flat one by minimal bone removal with a saw. The tibial bone defect is then assessed, and an appropriate-sized metal block is selected. A cutting guide for the block is assembled and a matching bone resection carried out. Care must be taken not to over-resect the bone, since the tibial blocks should be inserted in a tight manner. The positions of the central keels on the baseplate are prepared on the tibial surface.

The trial tibial component with the block and intramedullary stem is assembled and inserted. A trial reduction of the prosthesis is done, and alignment and stability with patellar tracking is assessed. After lavage, using pulsed normal saline and drying out of the prepared surfaces, blood and fat are kept out of the cement-prosthesis interface. The real components are assembled and cemented. We use one mix of PMMA cement with gentamicin to cement the tibial and femoral components separately.

Metal blocks of 10 mm + 10 mm or 10 mm + 5 mm are used on the medial aspect of the tibial component to compensate for bone defects. Both the tray and the block have a waffled surface that allows interdigitation of cement.

**Figure 3.**
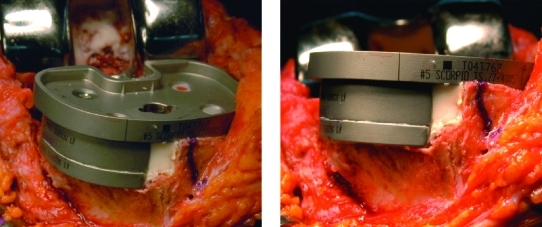
A. The first metal block is attached to the tibial tray with screws. B. After that, the next block is cemented to the first one.

**Figure 4.**
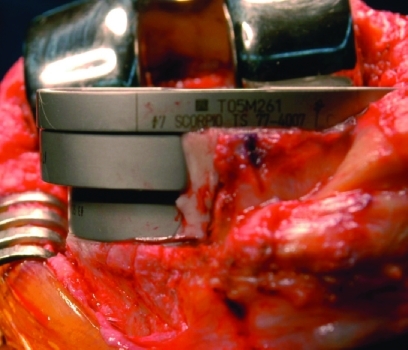
If the tibial blocks protrude over the cortical rim, downsized metal blocks may be available (another case).

The first block is attached to the tibial tray with screws. After that, the next block is cemented to the first one (Figure 3). The intramedullary stems on the tibial components are commonly used to protect the peripheral bone from stress. Finally, the real prosthesis with cemented tibial stem and with 10 mm + 10mm or 10 mm + 5 mm double metal augmentation are cemented into place. Downsized metal blocks can be used when the tibial blocks protrude over the cortical rim because of the natural taper of the proximal tibia (Figure 4).

Once the cement is cured and excess is removed, a polyethylene implant is inserted and the knee is reduced. Final checks of soft tissue tension, alignment, patellar tracking, and range of knee movements is made before wound closure.

A continuous passive motion is started within 24 h postoperatively, progressing slowly in flexion. Passive knee extension is encouraged by placing the patient's foot on a pillow while in bed. Weight bearing with the aid of crutches or a cane starts on the fifth or sixth postoperative day.

## Patients

Between 2004 and 2007, we carried out metal augmentation of tibial defects in 9 patients during either primary or revision knee arthroplasty, using the operative technique described. All the procedures were carried out by a single surgeon. Primary diagnosis were osteoarthritis in 4 cases, rheumatoid arthritis in 1, aseptic loosening in 2, and septic loosening in 1 case. Patients' mean age at operation was 65 (51–80) years. The mean follow-up period was 5 (2.5–6) years.

**Figure 5.**
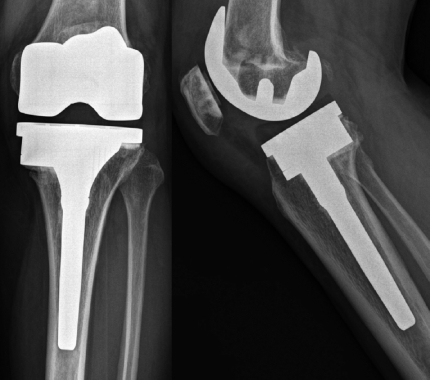
Immediate postoperative radiographs of knee replacement with double metal blocks.

**Figure 6.**
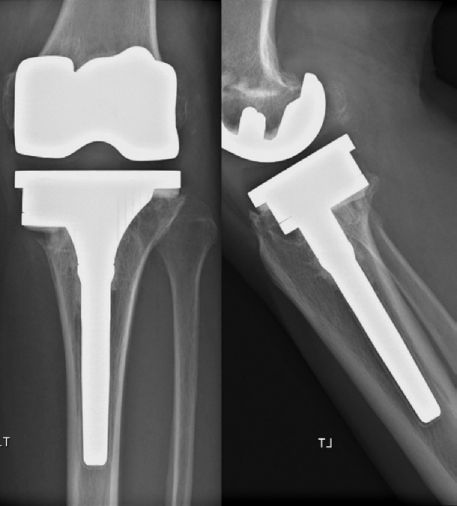
Postoperative radiographs at 5-year follow-up.

During the follow-up period, all the patients had a pain-free knee. The mean range of motion was 127° (120–135), and there was no radiographic evidence of prosthesis loosening or subsidence (Figures 5 and 6).

## Discussion

The indication for augmentation of bone defects in TKA is inability to achieve stability of the trial implants at the time of trial reduction. This generally occurs when 40% or more of the bone-implant interface is unsupported by host bone (Cuckler 2004). Treatment options for large tibial bone defects include polymethylmethacrylate cement, morselized or structural allograft, metal augments, and custom or hinge/tumor prostheses (Radnay and Scuderi 2006).

Excessive cement augmented by screws and mesh techniques has been abandoned because the construct is not mechanically sound, and may result in cement fragmentation and early failure of the replacement (Brooks et al. 1984, Brand et al. 1989, Ritter et al. 1993).

The technique for the application of an autograft in primary knee arthroplasty has been well described by Windsor et al. (1986). It is physiologically sound, cheap, and reproducible—and has the advantage of bone stock preservation (Toms et al. 2009). However, Laskin (1989) reported a 33% failure rate at 5-year follow-up of autografts in primary arthroplasty.

Larger defects may require allograft augmentation (Cuckler 2004, Engh and Ammeen 2007). However, allograft bone has the disadvantages that is difficult to achieve a proper fit with the host bone (Ries 1996) and that although they remain structurally intact, they are frequently not revascularized; new bone is laid down only in the periphery of a dead allograft (Parks and Engh 1997).

Custom implants can be used for large bone defects, and should theoretically provide the best fit and force transmission of any of the methods used to address bone deficiency (Brooks et al. 1984). However, they are expensive and often require considerable time to manufacture.

Modular implants with metal augmentation facilitate the treatment of bony defects (Patel et al. 2004, Fehring et al. 1996, Nelson et al. 2003, Pagnano et al. 1995). The advantage of modular metal augments is that they offer flexibility during the operation but still provide the surgeon with the ability to deal with defects of bone ranging from 5 to 10 mm in depth in various locations on the tibial plateau (Rand 1998). However, custom prostheses may be required for larger defects.

We have modified the operative method by attaching 2 blocks with the use of cement and screws to allow up to 20 mm of segmental bone defects to be restored. They can be applied quickly, allow intraoperative custom fabrication, and help restore an anatomic joint line. The technique is simple and has no learning curve.
